# Demonstration of polarization-insensitive spatial light modulation using a single polarization-sensitive spatial light modulator

**DOI:** 10.1038/srep09959

**Published:** 2015-07-06

**Authors:** Jun Liu, Jian Wang

**Affiliations:** 1 Wuhan National Laboratory for Optoelectronics, School of Optical and Electronic Information, Huazhong University of Science and Technology, Wuhan 430074, Hubei, China

## Abstract

We present a simple configuration incorporating a single polarization-sensitive phase-only liquid crystal spatial light modulator (LC-SLM) to facilitate polarization-insensitive spatial light modulation. The polarization-insensitive configuration is formed by a polarization beam splitter (PBS), a polarization-sensitive phase-only LC-SLM, a half-wave plate (HWP), and a mirror in a loop structure. We experimentally demonstrate polarization-insensitive spatial light modulations for incident linearly polarized beams with different polarization states and polarization-multiplexed beams. Polarization-insensitive spatial light modulations generating orbital angular momentum (OAM) beams are demonstrated in the experiment. The designed polarization-insensitive configuration may find promising applications in spatial light modulations accommodating diverse incident polarizations.

Spatial light modulator (SLM) is an important and useful device which can impose spatially varying modulation on light waves^1^. Recently, SLM has attracted increasing interest in miscellaneous applications, such as diffractive optics^2^, adaptive optics^3^,^4^,^5^, optical tweezers^6^, optical information processing^7^,^8^,^9^, and holographic projection^10^. The advantages employing SLM include high degree of flexibility, fast switching time, and good reconfigurablity and reproducibility.

The modulation mechanism of SLM relies on the method changing properties of the modulation material and the way altering the spatial field of an incident light beam. Various methods including mechanical, electro-optical, thermo-optical, magneto-optical effects can be used to change properties of the modulation material of an SLM, which interacts with an incident light and transforms its spatial field distribution. One of the most commonly used modulation mechanisms today is the electro-optical spatial light modulator containing liquid crystals as the modulation material. For liquid crystal spatial light modulator (LC-SLM), the optical properties of liquid crystals are modified by the applied electric field^11^. Typically, liquid crystals are birefringent due to their anisotropic nature with two refractive indices (i.e. the extraordinary refractive index *n*_*e*_ and the ordinary refractive index *n*_*o*_). A common criticism of LC-SLM is that it is polarization sensitive since only the refractive index and resultant phase shift of the extraordinary polarized light can be varied by the applied electric field^12^. However, polarization-insensitive operations are also highly desirable in some applications. For instance, linearly polarized beams with different polarization states and polarization-multiplexed beams are widely used in optical telecommunications systems^13^, where the usage of polarization-sensitive phase-only LC-SLM is considerably limited. In this scenario, a challengeable goal would be to develop a polarization-insensitive configuration while still using commonly available polarization-sensitive phase-only LC-SLM.

In this paper, we propose a simple configuration incorporating a single polarization-sensitive phase-only LC-SLM to facilitate polarization-insensitive spatial light modulation. We demonstrate polarization-insensitive spatial light modulations both for incident linearly polarized beams with different polarization states and polarization-multiplexed beams in the experiment.

## Results

### Concept of polarization-insensitive spatial light modulation

**Figure 1:**
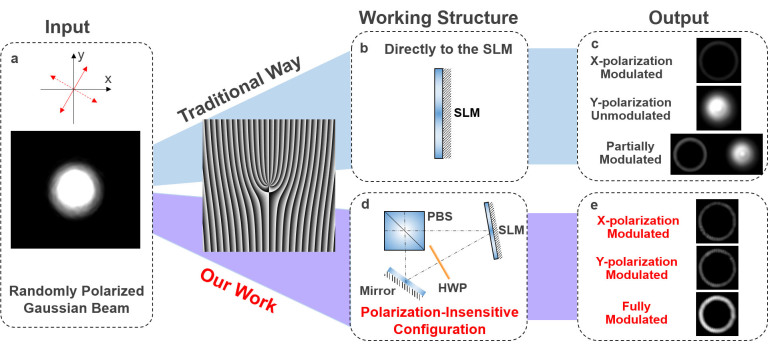
Concept and principle of polarization-insensitive spatial light modulation.

**Figure 2:**
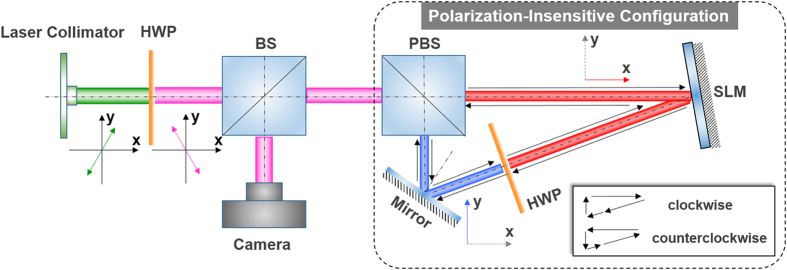
Experimental setup for polarization-insensitive spatial light modulation. HWP: half-wave plate. BS: non-polarization beam splitter. PBS: polarization beam splitter. SLM: spatial light modulator.

The concept and principle of the proposed configuration enabling polarization-insensitive spatial light modulation are illustrated in Fig. 1. For description simplicity, we assume that the polarization-sensitive phase-only LC-SLM only works for the x-polarization while no response to the y-polarization is expected. In general, when a randomly polarized Gaussian beam shown in Fig. 1(a) is delivered directly to the SLM shown in Fig. 1(b), the incident beam can be decomposed into two orthogonal polarizations, i.e. x-polarization and y-polarization, and only x-polarization is modulated by the SLM without touching the y-polarization. Hence, unmodulated y-polarization remains a Gaussian beam while modulated x-polarization becomes a newly converted beam, e.g. orbital angular momentum (OAM) beam having a spiral phase front and a doughnut intensity profile (a fork pattern loaded to the SLM), as shown in Fig. 1(c). In contrast, Fig. 1(d) illustrates the proposed polarization-insensitive configuration incorporating a polarization beam splitter (PBS), a polarization-sensitive phase-only LC-SLM, a half-wave plate (HWP), and a mirror in a loop structure. The output transmission port and reflection port of the PBS are along x-polarization and y-polarization, respectively. The fast axis (i.e. the axis through which the light travels faster) of the HWP is 45^o^ with respect to the x-polarization. Consequently, an incident randomly polarized Gaussian beam fed into the configuration is first split into x-polarization and y-polarization, which then propagate clockwise and counterclockwise around a loop configuration in Fig. 2, respectively. For the x-polarized Gaussian beam propagating clockwise, it is modulated by the SLM, the output of which rotates 90^o^ to be y-polarization after passing through the HWP. For the y-polarized Gaussian beam propagating counterclockwise, it is rotated 90^o^ and changed to x-polarization by the HWP, the output of which is also modulated by the SLM. So a single SLM accomplishes spatial light modulations for both two polarizations by running it in a bidirectional loop configuration assisted by PBS, HWP and mirror. After spatial light modulations, the light beams propagating clockwise and counterclockwise are combined again by the PBS to output spatially modulated randomly polarized beams. Shown in Fig. 1(e) is an example of polarization-insensitive OAM beam generation using the designed configuration. Both x-polarization and y-polarization are modulated to OAM beams.

### Experimental setup

Figure 2 shows the experimental setup for polarization-insensitive spatial light modulation. The key part of the setup depicted in the dashed box is the proposed polarization-insensitive configuration as illustrated in Fig. 1(d). The polarization of a collimated laser beam is adjusted by an HWP to offer variable input polarization to the polarization-insensitive configuration. A non-polarization beam splitter (BS) is used to serve as both input port delivering light beam to the configuration and output port reflecting light beam to the camera. In the polarization-insensitive configuration, the light path connecting PBS, SLM and HWP is x-polarized while the light path linking PBS, mirror and HWP is y-polarized. The HWP enables an exchange between x-polarization and y-polarization.

**Figure 3:**
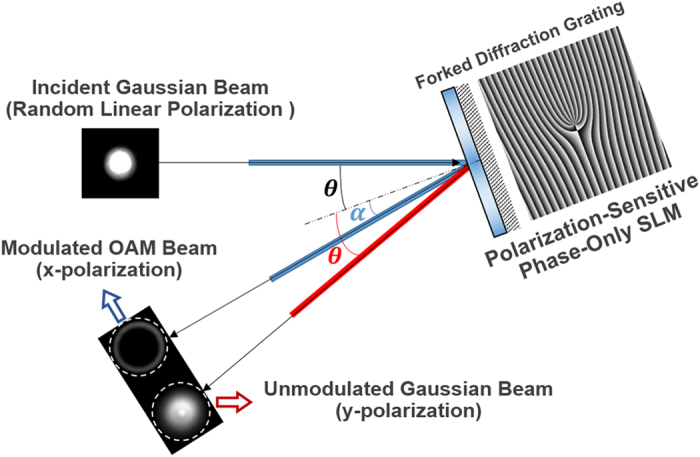
Illustration of the separation between the partially modulated x-polarization (OAM beam) and unmodulated y-polarization (Gaussian beam).

In the experiments, a forked diffraction grating (fork pattern) formed by a spiral phase distribution and a linear phase ramp is employed. The spiral phase distribution adds a spiral phase front to the incident Gaussian beam and converts it to an OAM beam^14^. The linear phase ramp outputs the generated OAM beam along the first diffraction order. As illustrated in Fig. 3, when a randomly polarized Gaussian beam is projected onto the SLM loaded with a forked diffraction grating (incident angle: θ relative to the normal of the SLM), the decomposed x-polarization is modulated to be an OAM beam which outputs along the first-order diffraction direction (α with respect to the normal of the SLM). By contrast, the decomposed y-polarization is unmodulated and directly reflected by the SLM as a “mirror” along the regular reflection direction (θ relative to the normal of the SLM). Hence, the partially modulated x-polarization (OAM beam) and unmodulated y-polarization (Gaussian beam) separate with each other. When employing the proposed polarization-insensitive configuration, not only the decomposed x-polarization but also the decomposed y-polarization which is first rotated to the x-polarization, are effectively modulated by the SLM and then delivered along the first-order diffraction direction, resulting in the overlap of two modulated polarizations.

### Different linearly polarized beams directly modulated by the SLM

**Figure 4:**
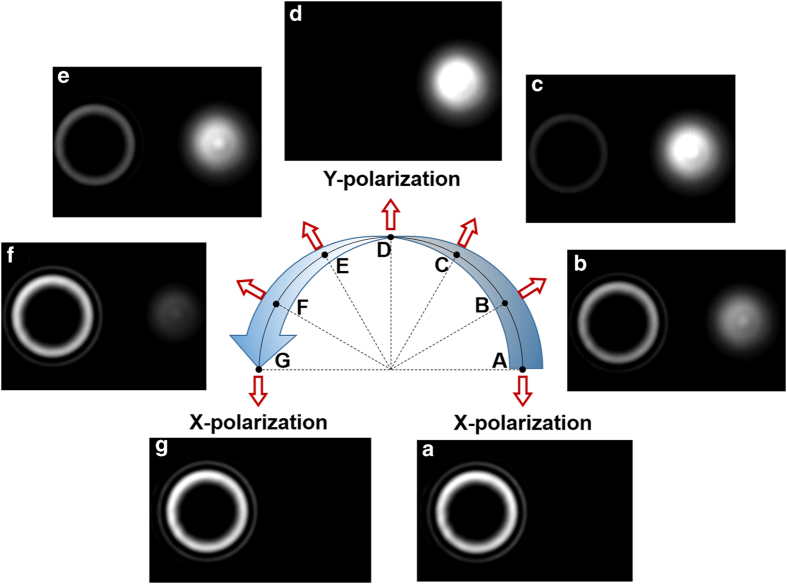
Intensity profiles for output beams under different angles between the incident polarization and x-polarization of (**a**) 0, (**b**) 30, (**c**) 60, (**d**) 90, (**e**) 120, (**f**) 150 and (**g**) 180 degree (direct spatial modulation by a polarization-sensitive SLM).

We first study the spatial light modulation when different linearly polarized beams are directly modulated by the SLM. The dashed box in Fig. 2 is simply replaced by a polarization-sensitive phase-only LC-SLM. The camera is moved to the path of the reflected beam from the SLM. Fig. 4(a)–(g) depict measured intensity profiles for different reflected beams from the SLM under different angles between the incident polarization and x-polarization of 0, 30, 60, 90, 120, 150 and 180 degree, respectively. One can clearly see interesting phenomena as follows: 1) Two separated beams are observed with one modulated x-polarization OAM beam and the other unmodulated y-polarization Gaussian beam, which are in agreement with those illustrated in Fig. 3. 2) With the increase of the angle from 0 to 90 degree, the decomposed x-polarization decreases while y-polarization increases, resulting in the decrease of the modulated OAM beam while increase of the unmodulated Gaussian beam. When further increasing the angle from 90 to 180 degree, opposite evolution trend is observed, i.e. modulated OAM beam increases while unmodulated Gaussian beam decreases.

**Figure 5:**
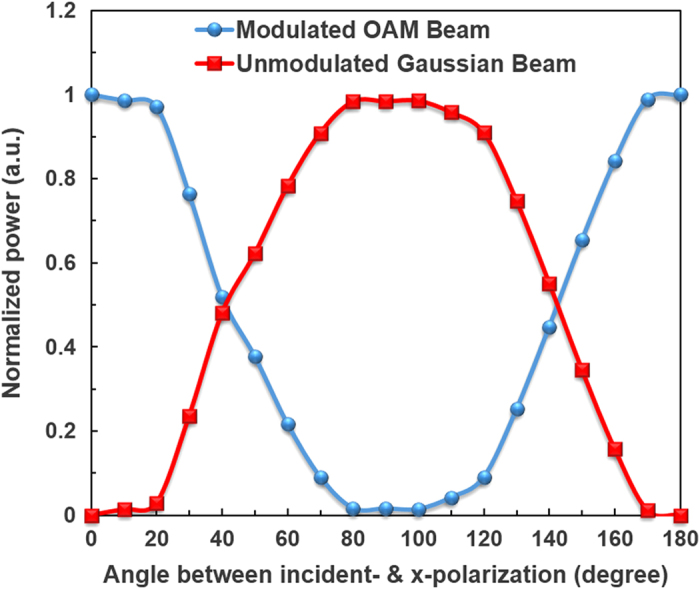
Normalized power for modulated x-polarization OAM beam and unmodulated y-polarization Gaussian beam versus angle between the incident polarization and x-polarization (direct spatial modulation by a polarization-sensitive SLM).

Figure 5 plots measured normalized power for modulated x-polarization OAM beam and unmodulated y-polarization Gaussian beam as a function of the angle between the incident polarization and x-polarization. The normalized power is defined as the measured power divided by the total power of modulated x-polarization OAM beam and unmodulated y-polarization Gaussian beam. The obtained results agree well with the observed intensity profiles shown in Fig. 4. It is noted that only modulated x-polarization OAM beam is achieved under angles of 0 and 180 degree while only unmodulated y-polarization Gaussian beam is obtained under angle of 90 degree. For other angles from 0 to 180 degree, both modulated x-polarization OAM beam and unmodulated y-polarization Gaussian beam are observed.

### Polarization-insensitive spatial light modulation

**Figure 6:**
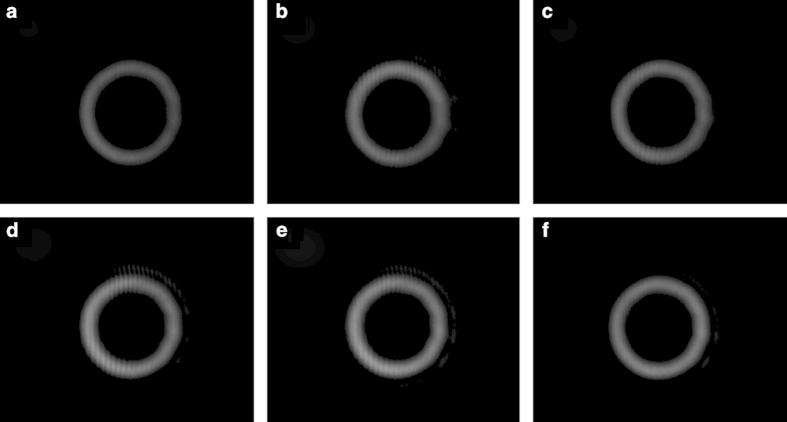
Intensity profiles for output beams under different angles between the incident polarization and x-polarization of (**a**) 0, (**b**) 40, (**c**) 90, (**d**) 120, (**e**) 140 and (**f**) 180 degree (spatial modulation by the polarization-insensitive configuration).

We then study the polarization-insensitive spatial light modulation by employing the polarization-insensitive configuration in the setup as shown in Fig. 2. Shown in Fig. 6 are measured intensity profiles for different output beams (reflected from BS) under different angles between the incident polarization and x-polarization of 0, 40, 90, 120, 140 and 180 degree, respectively. It can be clearly seen that both decomposed x-polarization and y-polarization are modulated to be “doughnut” shape OAM beams which overlap with each other. No distinct change is observed as varying the polarization of incident linearly polarized Gaussian beam.

**Figure 7:**
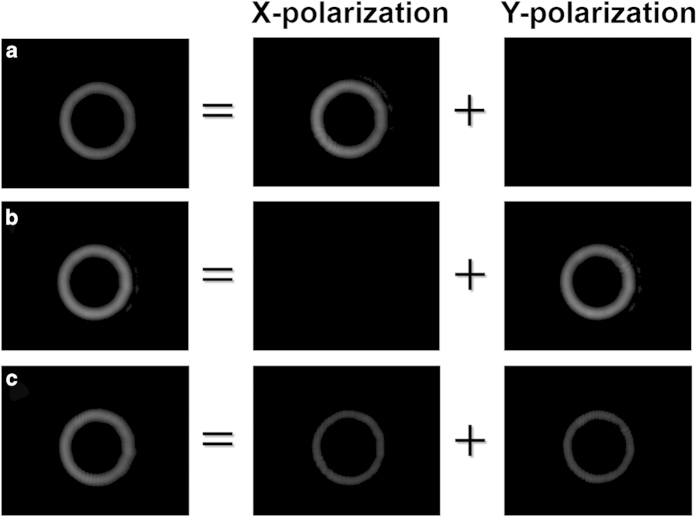
Intensity profiles for different output beams and their x- and y-polarization components when the incident Gaussian beam is x-polarization, y-polarization and 45°-polarization (spatial modulation by the polarization-insensitive configuration).

To further show the ability of spatial light modulation for both x-polarization and y-polarization, we put a polarizer before the camera which can extract the x-polarization and y-polarization components of output beams. Fig. 7(a)–(c) depict measured intensity profiles for different output beams (reflected from BS) and their x- and y-polarization components when the incident Gaussian beam is x-polarization, y-polarization and 45°-polarization, respectively. The 1st, 2nd and 3rd columns represent overall output beam without using polarizer before camera, x-polarization component and y-polarization component using polarizer before camera, respectively. For x-polarized incident Gaussian beam, y-polarization component of output beam is zero while x-polarization component almost the same as the overall output beam. For y-polarized incident Gaussian beam, x-polarization component of output beam is zero while y-polarization component similar to the overall output beam, showing the successful spatial modulation of y-polarization. For 45°-polarized incident Gaussian beam, the x-polarization and y-polarization components of output beam are almost equal to each other.

**Figure 8:**
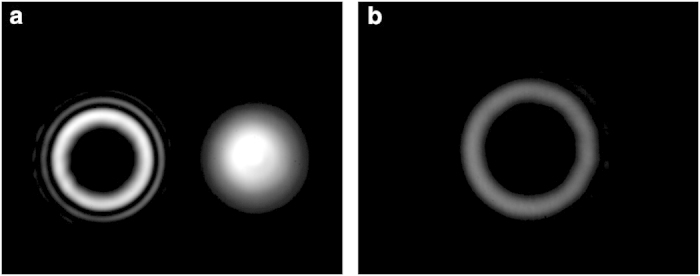
Intensity profiles for output beams with polarization-multiplexed incident Gaussian beams. (**a**) Direct spatial modulation by a polarization-sensitive SLM. (**b**) Spatial modulation by the polarization-insensitive configuration.

We finally study the spatial light modulation by employing polarization-multiplexed incident Gaussian beams. The x-polarization and y-polarization are from separate laser sources. When directly using a SLM, similar result to Fig. 4, i.e. two separated beams with one modulated x-polarization OAM beam and the other unmodulated y-polarization Gaussian beam, is observed as shown in Fig. 8(a). When using polarization-insensitive configuration, as shown in Fig. 8(b), it is found that similar result to Fig. 6 is obtained, i.e. both x-polarization and y-polarization are modulated to be OAM beams overlapping with each other.

The obtained results shown in Figs. 6, 7, 8 confirm the successful implementation of polarization-insensitive spatial light modulation, i.e. it is possible to construct a polarization-insensitive configuration with its key component of a polarization-sensitive spatial light modulator. The presented polarization-insensitive spatial light modulation might find useful and wide applications when linearly polarized beams with different polarization states, polarization-multiplexed beams, or even randomly polarized beams are applied.

## Discussion

We propose and demonstrate a simple configuration enabling polarization-insensitive spatial light modulation using a single polarization-sensitive phase-only LC-SLM. The designed polarization-insensitive configuration is constructed by a PBS, a polarization-sensitive phase-only LC-SLM, an HWP and a mirror. We compare the results of spatial light modulations when directly using a polarization-sensitive LC-SLM and employing the polarization-insensitive configuration. For incident linearly-polarized beams with different polarization states and polarization-multiplexed beams, partial modulation by a direct LC-SLM while full modulation by the polarization-insensitive configuration are demonstrated in the experiment. Moreover, polarization-insensitive spatial light modulation converting Gaussian beams to OAM beams are realized in the experiment. It is expected that the presented polarization-insensitive configuration might see its attractive applications when diverse incident polarizations are applied to the spatial light modulations.

## Methods

Several methods for achieving polarization-insensitive spatial light modulation have been discovered, e.g. using a double passage through a nematic LC cell^15^, using a blue-phase liquid crystal over silicon device^16^, and using a thin polymer-separated double-layered structure^17^. Here the proposed polarization-insensitive configuration is formed by a polarization beam splitter, a polarization-sensitive phase-only LC-SLM, a half-wave plate, and a mirror in a loop structure.

To demonstrate the functionality of the proposed configuration, a spiral phase distribution is loaded on the SLM to convert the incident Gaussian beam to an OAM beam. A linear phase ramp is employed to distinguish the modulated beam and the unmodulated one spatially. Therefore, a forked diffraction grating formed by a spiral phase distribution and a linear phase ramp generates OAM beam along the first diffraction order.

## Additional Information

**How to cite this article**: Liu, J. and Wang, J. Demonstration of polarization-insensitive spatial light modulation using a single polarization-sensitive spatial light modulator. *Sci. Rep.* **5**, 9959; doi: 10.1038/srep09959 (2015).

## References

[b1] Maurer C., Jesacher A., Bernet S. & Ritsch-Marte M. What spatial light modulators can do for optical microscopy. Laser Photon. Rev. 5, 81–101 (2011).

[b2] Márquez A., Iemmi C., Campos J., Escalera J. & Yzuel M. Programmable apodizer to compensate chromatic aberration effects using a liquid crystal spatial light modulator. Opt. Express 13, 716–730 (2005). 19494932 10.1364/opex.13.000716

[b3] Dou R. & Giles M. K. Closed-loop adaptive-optics system with a liquid-crystal television as a phase retarder. Opt. Lett. 20, 1583–1585 (1995). 19862090 10.1364/ol.20.001583

[b4] Love G. D. Wave-front correction and production of Zernike modes with a liquid-crystal spatial light modulator. Appl. Opt. 36, 1517–1520 (1997). 18250829 10.1364/ao.36.001517

[b5] Laude V. Twisted-nematic liquid-crystal pixelated active lens. Opt. Commun. 153, 134–152 (1998).

[b6] Grier D. G. A revolution in optical manipulation. Nature 424, 810–816 (2003). 12917694 10.1038/nature01935

[b7] Karim M. A. & Awwal A. A. S. Electrooptic displays for optical information processing. Proc. IEEE 84, 814–827 (1996).

[b8] Yu F. & Lu X. A real-time programmable joint transform correlator. Opt. Commun. 52, 10–16 (1984).

[b9] Liu H.-K., Davis J. A. & Lilly R. A. Optical-data-processing properties of a liquid-crystal television spatial light modulator. Opt. Lett. 10, 635–637 (1985). 19730510 10.1364/ol.10.000635

[b10] Mok F., Diep J., Liu H.-K. & Psaltis D. Real-time computer-generated hologram by means of liquid-crystal television spatial light modulator. Opt. Lett. 11, 748–750 (1986). 19738748 10.1364/ol.11.000748

[b11] Igasaki Y. *et al.* High efficiency electrically-addressable phase-only spatial light modulator. Opt. Rev. 6, 339–344 (1999).

[b12] Hu L. *et al.* A polarization independent liquid crystal adaptive optics system. J. Opt. 12, 045501 (2010).

[b13] Wang J. *et al.* Terabit free-space data transmission employing orbital angular momentum multiplexing. Nature Photon. 6, 488–496 (2012).

[b14] Li S. *et al.* Demonstration of simultaneous 1-to-34 multicasting of OFDM/OQAM 64-QAM signal from single gaussian mode to multiple orbital angular momentum (OAM) modes. Asia Communications and Photonics Conference (Optical Society of America, 2013), Beijing, China, postdeadline paper AF2E.5 (2013).

[b15] Love G. D. Liquid-crystal phase modulator for unpolarized light. Appl. Opt. 32, 2222–2223 (1993). 20820371 10.1364/AO.32.002222

[b16] Hyman R. M., Lorenz A., Morris S. M. & Wilkinson T. D. Polarization-independent phase modulation using a blue-phase liquid crystal over silicon device. Appl. Opt. 53, 6925–6929 (2014). 25322400 10.1364/AO.53.006925

[b17] Lin Y. H. *et al.* Polarization-independent liquid crystal phase modulator using a thin polymer-separated double-layered structure. Opt. Express 13, 8746–8752 (2005). 19498907 10.1364/opex.13.008746

